# Identification of Putative Olfactory Genes from the Oriental Fruit Moth *Grapholita molesta* via an Antennal Transcriptome Analysis

**DOI:** 10.1371/journal.pone.0142193

**Published:** 2015-11-05

**Authors:** Guangwei Li, Juan Du, Yiping Li, Junxiang Wu

**Affiliations:** 1 Key Laboratory of Plant Protection Resources and Pest Management (Northwest A&F University), Ministry of Education, Yangling, Shaanxi, People’s Republic of China; 2 Key Laboratory of Applied Entomology, Northwest A&F University, Yangling, Shaanxi, People’s Republic of China; Biogen Idec, UNITED STATES

## Abstract

**Background:**

The oriental fruit moth, *Grapholita molesta*, is an extremely important oligophagous pest species of stone and pome fruits throughout the world. As a host-switching species, adult moths, especially females, depend on olfactory cues to a large extent in locating host plants, finding mates, and selecting oviposition sites. The identification of olfactory genes can facilitate investigation on mechanisms for chemical communications.

**Methodology/Principal Finding:**

We generated transcriptome of female antennae of *G*.*molesta* using the next-generation sequencing technique, and assembled transcripts from RNA-seq reads using Trinity, SOAPdenovo-trans and Abyss-trans assemblers. We identified 124 putative olfactory genes. Among the identified olfactory genes, 118 were novel to this species, including 28 transcripts encoding for odorant binding proteins, 17 chemosensory proteins, 48 odorant receptors, four gustatory receptors, 24 ionotropic receptors, two sensory neuron membrane proteins, and one odor degrading enzyme. The identified genes were further confirmed through semi-quantitative reverse transcription PCR for transcripts coding for 26 OBPs and 17 CSPs. OBP transcripts showed an obvious antenna bias, whereas CSP transcripts were detected in different tissues.

**Conclusion:**

Antennal transcriptome data derived from the oriental fruit moth constituted an abundant molecular resource for the identification of genes potentially involved in the olfaction process of the species. This study provides a foundation for future research on the molecules involved in olfactory recognition of this insect pest, and in particular, the feasibility of using semiochemicals to control this pest.

## Introduction

The sensitive olfactory system plays a predominant role in insect behavior, such as seeking host plants, finding mates, selecting oviposition sites, recognizing kins, and escaping from predators and toxic compounds [1]. Antennae are specialized organs of insect for chemical sensing, especially for olfaction. The surface of antennae is covered with different types of sensilla, which are a specialized hair-like, multi-pore structures in which olfactory receptor neurons (ORNs) extend dendrites into the antennal lymph where peripheral olfactory signal transduction occur [2]. ORNs can recognize relevant volatiles and generate an electrical impulse that is transported to the primary olfactory center in the antennal lobe [3]. Within the sensilla-ORN structure, a number of gene families are involved in different steps in signal transduction pathways, such as the genes encoding odorant binding proteins (OBPs), chemosensory proteins (CSPs), odorant receptors (ORs), ionotropic receptors (IRs), sensory neuron membrane proteins (SNMPs), and odorant degrading enzymes (ODEs) [4].

OBPs belong to a group of small water-soluble proteins that are secreted by the accessory cells around the ORNs and impregnated in the sensilla lymph [5]. OBPs are considered to be the first group of proteins that participate in the olfactory signal transduction pathway in insects, which can selectively transport hydrophobic odorant molecules through the sensillum lymph to the surface of ORNs as the odor molecules diffuse through the pores on sensilla [6]. Like OBPs, the CSPs are another class of hydrophilic proteins that are enriched in the sensillum lymph. However, its function in olfactory transduction and non-olfactory procedures remains largely unknown [7]. ORs are embeded in the dendrite membrane of ORNs in the antennae, and play a central role as a bio-transducer in chemosensory signal transduction [8]. In insects, it is generally believed that ORs function as heterodimers, with highly conserved and broadly expressed protein (originally called Or83b but now with the generic name ORCO [9]) serve as a ligand-gate channel with a various partner (OrX) that can distinctly determine ligang-binding specificity [10]. In addition, ORs could recognize odorants and therefore are also involved in odor recognition [11]. Typically, there are three transmembrane domains and a bipartite ligand-binding domain with two lobes in IRs [12]. IRs act in complex of three subunits, which can be composed of individual odor-specific receptors, and one or two of the broadly expressed coreceptors (IR8a, IR25, and IR76b) in one IR-expressing neuron [11].

The development of Next Generation Sequencing (NGS) technologies has greatly improved the efficiency and speed of gene discovery in recent years [13]. *De novo* assembly of transcripts provides a workable solution to transcriptome analysis. At present, a lot of *de novo* transcriptome assemblers available are designed for Roche 454, Illuminia Solexa, and SOLID. SOAPdenovo, SOAPdenovo-Trans, Velvet Oases, ABySS, trans-ABySS, and trinity have been successfully applied to *de novo* transcriptome assembly form short-read RNA-Seq data of model and non-model organisms [3,14–18].

The oriental fruit moth *Grapholita molesta* Busck is an economically important oligophagous pest species of stone and pome fruits throughout the world, causing substantial losses in fruit yields [19]. Peach (*Prunus persica* L) is considered the primary host, and pear (*Pyrus communis* L.) and apple (*Malus domestica* L.) are secondary hosts [20,21]. In some parts of their geographic range, the adult can migrate from peach orchards to pear or apple orchards by detecting and following changes of volatile components emitted by these host plants [22]. A lot of pests with multiple generations, such as *G*.*molesta*, that can annually survive and reproduce on different hosts, are confronted with hight-variability in the volatile blends emitted by different host plant species at specific periods, as well as by the same host plant species across a growing season [23]. One way to adapt these variations in host plant volatile blends is to respond to a specific set of compounds common to all host plants [24]. A three-component mixure of (Z)-3-hexen-1-yl acetate, benzaldehyde and (Z)-3-hexen-1-ol elicited a similar attractant effect on *G*.*molesta* as the natural blend from peach shoots [25]. The mixture of (Z)-3-hexenyl acetate: (Z)-β-ocimene: (E)-β-farnesene in the proportion 1:2:2 can attract mated *G*. *molesta* males [26]. Small amounts of benzonitrile can convert an unattractive four-compound mixture ((Z)-3-hexen-1-yl acetate, (Z)-3-hexen-1-ol, benzaldehyde, and (E)-2-hexenal, ratio 69.84:14.64:13.26:2.26) to a bioactive five-compound mixture that is attractive to mated *G*.*molesta* females as good as natural blends [27]. Volatiles blends from the various attractive stages of peach and pear shared a common set of five aldehydes, suggesting the C6-C10 aliphatic aldehydes play a key role in *G*.*molesta* females attraction to host plants [28]. Butyl hexanoate makes up about 10% of the total volatiles emitted from peach shoots and ripe pears. Mated *G*. *molesta* females are attracted to butyl hexanoate at intermediate dosages [29]. However, limited information is available on the olfactory recognition mechanism for host plant’s volatiles at molecular levels.

The monitoring of *G*.*molesta* mainly lies on pheromone trapping of males. However, the flight performance of this species exhibits remarkable differences between males and females. The proportion of long-flying females was three to six times greater than males, and gravid females can be considered to be the main colonists [30]. In the field, female moths have the capacity to make inter-orchard flights [31], causing a serious threat to pear or apple orchards especially in the vicinity of peach crops. Female moths are more easily to tolerate a modulation of the ratios of volatile compounds with distinct threshold values [32]. Therefore, identification of a wider range of olfactory genes of female moths will enable a better understanding of the mechanisms of olfactory recognition at the molecular level, which could ultimately lead to the development of new environment-friendly control strategies.

To identify genes likely involved in olfaction in species with no sequenced genome available like *G*. *molesta*, we sequenced and analyzed an antennal transcriptome of adult females using Illumina Miseq sequencing. We reported here the identification of 28 OBP genes, 17 CSP genes, 48 OR genes, 24 IR genes, four GR genes, two SNMP genes, and one ODE gene in the female antennal transcriptome.

## Methods

### Insect rearing

*G*. *molesta* in all experiments were obtained from a laboratory colony maintained at the College of Plant Protection, Northwest A&F University, Yangling, Shaanxi, China. The colony of *G*. *molesta* has been maintained for more than 60 generations in the laboratory. Larvae were reared on artificial diet at 25 ± 1°C, 70 ± 5% RH under a photoperiod of 15:9 (L:D). After pupation, male and female pupae were placed in separate glass tubes and maintained in the conditions described previously. The adults were fed with a cotton swab dipped in 5% honey solution and changed daily. Antennae of 3–4 day-old female moths were dissected after eclosion, immediately frozen in liquid nitrogen, and stored at –80°C until RNA was extracted.

### Extraction of total RNA

Frozen antennae were immediately transferred into a 1.5 mL Eppendorf tube immersed in liquid nitrogen and ground with a pestle. Total RNA was extracted using RNAiso Plus Total RNA extraction reagent (TaKaRa, Shiga, Japan) following the manufacturer’s instructions. The residual genomic DNA in total RNA was removed by DNase I (MBI Fermentas, Glen Burnie, MD, USA). Total RNA was dissolved in RNase-free water and RNA integrity was measured using Agilent 2100 bioanalyzer (Quantifluor-ST fluorometer, Promega, E6090). The high quality RNA (RIN number: 9.3) was used for cDNA library construction and Illumina sequencing.

### Sequencing

Poly (A) mRNA was isolated from 12 μg of total RNA extracted from approximately 1200 antennae of 3–4 days-old adult female moths using the PolyA+Tract mRNA Isolation System (Illumina, San Diego, CA), and further purified using the RNeasy MinElute Clean up Kit (Qiagen, Hilden, Germany) following the manufacturer’s protocol. Fragmentation buffer was added to cleave mRNA into short fragments, and then, these fragments were used to synthesize first-strand cDNA using random hexamer primers, which was further transformed into double stranded cDNA with RHase H and DNA polymerase I. A paired-end library was constructed from the cDNA synthesized using the Genomic Sample Prep Kit (Illumina). Fragments larger than 375 bp were purified with QIAquick PCR Extraction Kit (Qiagen), end-repaired, and linked with sequencing adapters. AMPureXP beads were used to remove the unsuitable fragments, and then, the sequencing library was constructed with PCR amplification. After being validated using Pico green staining (Quant-iT PicoGreen dsDNA Assay Kit, Invitrogen, P7589) and fluorospectrophotometry, and quantified using Agilent 2100 (Quantifluor-ST fluorometer, Promega, E6090)), the library was sequenced using Illumina Miseq platform (Shanghai Personal Biotechnology Cp., Ltd. Shanghai, China). For subsequent analysis, 1/2 run data was generated.

### Unigene generation

Raw reads were filtered using a stringent process and subsequent *de novo* assembly. The reads were screened from the 39 to 59 to trim the bases with a quality score of Q<20 using 5 bp windows, and the reads with final length less than 50 bp were removed. In order to accurately discover and reduce false positive olfactory gene detection, we evaluate the performance of *de novo* transcriptome assembly using SOAPdenovo-Trans, Trans-ABySS, and Trinity form short-read RNA-Seq data of *G*.*molesta* antennae. The *de novo* transcriptome assembly was further analyzed using DETONATE (*de novo* transcriptome RNA-seq assembly with or without the truth evaluation) [33] and Transrate (http://hibberdlab.com/transrate/index.html). All derived transcript sequences were used to search NCBI non-redundant (NR) database (ftp://ftp.ncbi.nlm.nih.gov/blast/db/) with the BLASTn program (E-value,1E-5), and the top-hit transcripts were selected as unigenes. For the unigenes that failed to be aligned with any sequence in the databases, the software GetORF was used to predict their open reading frames (ORFs) and ascertain their coding orientations with default settings.

### Gene identification and functional annotation

The annotation of all derived sequences were executed using the BLASTX program against the NCBI non-redundant database (NR) and SwissProt protein sequences with e-value<1e-5. The BLASTX results were then imported into Blast2GO suite for GO Annotation. Open reading frames (ORFs) of the unigenes were predicted using ORF Finder (http://www.ncbi.nlm.nih.gov/gorf/gorf.html) based on the results given by BLASTX. ClustalX version 1.83 and MAFFT version 6.0 (http://mafft.cbrc.jp/alignment/server/) were used to conduct multiple sequence alignments. Signal peptides of the protein sequences were identified using SignalP 4.0 (http://www.cbs.dtu.dk/services/SignalP/) with default parameters. The transmembrane-domains (TMDs) of annotated genes were predicted using TMHMM version 2.0 (http://www.cbs.dtu.dk/services/TMHMM), while the ORFs were translated to amino acid sequences using ExPASy (http://www.expasy.org/translate/).

### Phylogenetic analyses

To verify the annotation of the candidate olfaction genes and to search for orthologs, phylogenetic analyses were conducted among *G*. *molesta* and other species with close genetic relationships. The species we selected is all belong to Lepidoptera. The genomes of *Bombyx mori* and *Danaua plexippus* have been published. The transcriptomes of *Agrotis ypsilon*, *Heliothis virescens*, *Heliothis armigera*, *Cydia pomonella* and *Manduca sexta* concentrated on olfactory genes have been well studied, as well as the function of these genes [16,18,34–37]. The OBP data set contained 28 sequences which are identified as candidate GmolOBPs, four sequences from *C*. *pomonella* [34], nine sequences from *D*. *plexippus* [35], 16 sequences from *H*. *virescens* [36], 13 sequences from *M*. *sexta* [37], 20 sequences from *H*. *armigera* [20], and 13 sequences from *A*. *ipsilon* [18]. All together, the OBP data set contained 103 sequences. The CSP data set contained 77 sequences, including 16 sequences from *B*. *mori* [7], four sequences from *C*. *fumiferana* [38], 17 sequences from *H*. *armigera* [39], nine sequences from *H*. *virescens* [36], 14 sequences from *S*. *littoralis* [15], and 17 sequences which are identified as candidate GmolCSPs. The OR and GR data set contained OR (or GR) sequences identified in Lepidoptera (one from *M*. *sexta* [14], 41 from *C*. *pomonella* [16], five from *S*. *littoralis* [15] and 69 from *B*. *mori* [40]). The OR data set contained a total of 168 sequences. In the IR data set, 24 sequences of candidate IRs from *G*. *molesta* were added to the number of sequences identified in *B*. *mori* [40], *M*. *sext* [14], *C*. *pomonella* [16], and *S*. *littoralis* [15]. Since IRs are more conserved than ORs among insects, IR sequences from non-Lepidoptera species (*D*. *melanogaster* [11], *A*. *mellifera* [41] and *T*. *castaneum*) were also included in the data set, and the final data set contained 175 sequences. Amino acid sequences of proteins used in building the phylogenetic tree are listed in S1 File. Amino acid sequences were aligned using MAFFT version 6.0, while the unrooted trees were constructed by the neighbor-joining method, with observed correction of distances, as implemented in Seaview v.4 software. The node support was assessed using a bootstrap procedure base on 1000 replicates, and the tree was drawn using Adobe Photoshop CS5.

### Expression analysis of the candidate OBPs and CSPs by semi-quantitative reverse transcription PCR

To confirm and compare the tissue expression of putative GmolOBPs and GmolCSPs identified from the transcriptome, semi-quantitative reverse transcription PCR was performed using cDNAs template prepared from male antennae, female antennae, and remaining bodies (without antennae) of the moth. Each experiment contained two biological repeats, three technical duplications, and controls were PCR with no template. Total RNA was extracted as described previously, treated with DNAse (RQ1, Promega, Madison, WI, USA), and corresponding cDNAs were synthesized using the First Strand cDNA Synthesis Kit (TaKaRa, Shiga, Japan) following the recommended protocol. Primers were designed using the Primer Premier 5 software and sequences are available in S1 Table. PCR was performed with GeneAmp PCR system 9700 under the general 3-step amplification of 95°C for 5 min, followed by 30 cycles of 95°C for 30 s, 50–60°C for 30 s; 72°C for 30 s, and final extension of 72°C for 10 min. The PCR cycle-numbers were adjusted respectively for each gene. For most chemosensory genes, cycle-numbers were within the range of 30 to 35, but for some genes with high levels of expression, cycle-numbers were reduced to 25. PCR products were analyzed on 1.2% agarose gels electrophoresis and verified by direct DNA sequencing.

## Results

### Sequencing and *de novo* assemblies

A total of 5.6 million raw reads (average read length 251 bp) were obtained from the libraries of female antennae. After removing low quality, adaptor, and contaminating sequence reads and reads shorter than 50 bp, about 5.2 million clean-reads comprised the 2.2 gigabases were generated. In total, 114263, 79209 and 71086 transcripts, with the mean length of 630, 510, and 619 bp, were obtained from assembled with Trinity, SOAPdenovo-trans and Trans-Abyss (Table 1). The raw data from IIIumina Miseq sequencing was deposited in the NCBI Short Read Archive (SRA) database with accession number SRR1424578. The gene lengths, reads number of each unigene, and the abundance of the unigenes based on reads were integrated in S2 Table.

**Table 1:** Assembly summary of *G*. *molesta* antenna transcriptome using three different assemblers.

Assemblers	Total Length (bp)	Transcripts No.	Max Length (bp)	Mean Length (bp)	N50	>1K reads No.
Trinity	71953993	114263	24397	630	996	17955
SOAPdenovo-trans	40414085	79209	21711	510	751	8883
Abyss-trans	43992300	71086	26749	619	940	10766

### Quality assessment of *de novo* transcriptome assemblies

**Fig 1:**
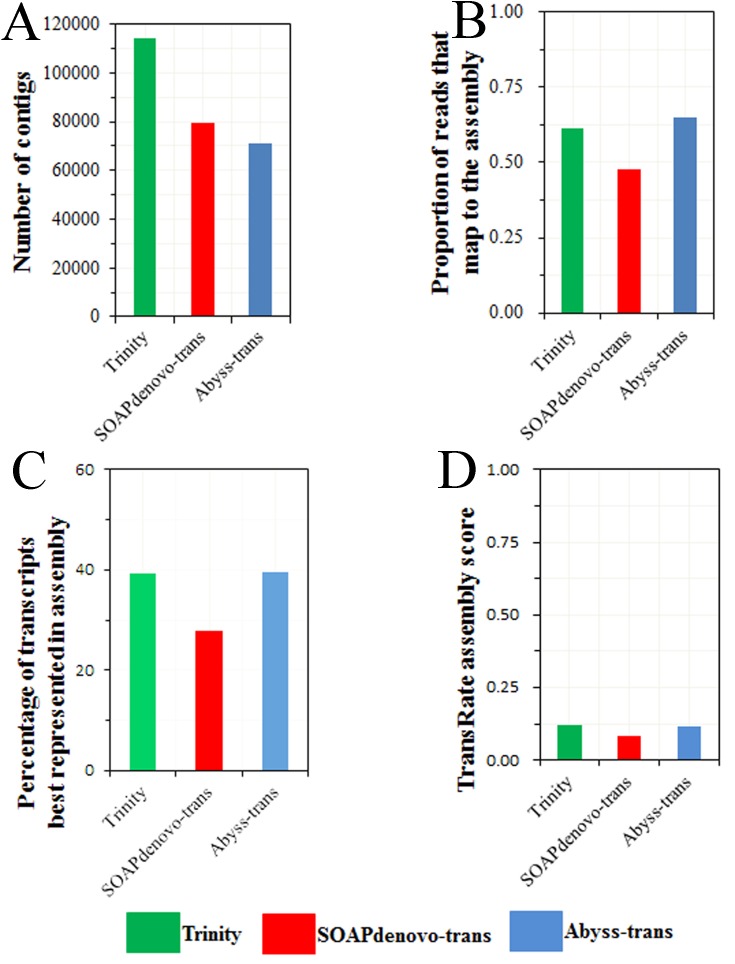
TransRate assembly scores. (A) Number of contigs for three representative assemblies from RNA-seq data of *G*.*molesta*. (B) Proportion of reads that map to each assembly. (C) Percentage of transcripts best represented in the assembly. (D) Final TransRate assembly scores for the three different assemblies.

Trinity assembly produced the most transcripts, the longest transcripts in average and the largest N50, followed by Abyss-trans, while SOAPdenovo-trans yielded the worst in very category (Table 1). Among the three different *de novo* assemblers, we obtained the same number transcripts annotated to putative olfactory genes by the search against the non-redundant protein database. Most of the cover percent transcripts annotated to olfactory genes were greater than 70% among the three assemblers (S3 Table). We evaluated the transcriptome assembly generated using DETONATE and TransRate. The results showed that calculated TransRate assembly scores of Trinity and Abyss-trans had no much difference, but greater than score of SOAPdenovo-trans (Fig 1), revealing the Trinity and Abyss-trans conducted more accurately and completely on individual contigs level than SOAPdenovo-trans. The likelihood score, which the dominant term in the RSEM-EVAL score, was much higher in Trinity and Abyss-trans assembly than SOAPdenovo-Trans (Table 2), indicating that the Trinity and Abyss-trans are more accurate in assembly-level than SOAPdenovo-trans. Overall, Trinity is the most suitable software for *de novo* RNA-seq assembly for *G*.*molesta* without sequenced genomes.

**Table 2:** RSEM-EVAL evaluating *de novo* assemblies from Trinity, SOAPdenovo-Trans and Abyss-trans.

Assembler	Likehood score	Prior score	BIC score	RSEM-EVAL score
Trinity	-2590055043	-99749414	-887936	-2690692395
SOAPdenovo-trans	-2978072059	-55965628	-615534	-3034653222
Abyss-trans	-2618491605	-60986277	-552411	-2680030294

RSEM-EVAL was run on each assembly and the likelihood, prior, BIC and total RSEM-EVAL scores were recorded. BIC, Bayesian information criterion.

### Gene identification and functional annotation

**Fig 2:**
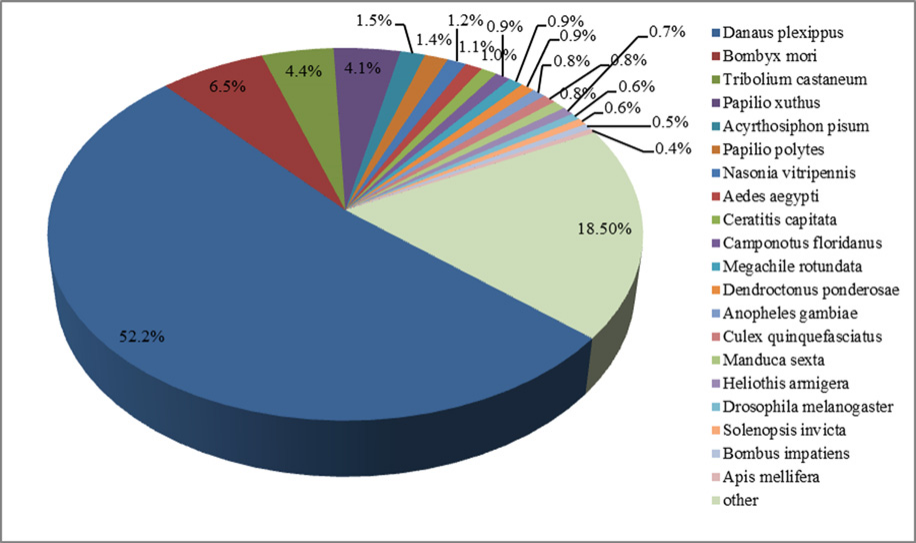
Top 20 best hits of the BLASTx results. All *G*. *molesta* antennal unigenes were used in BLASTX search in NR database.

**Fig 3:**
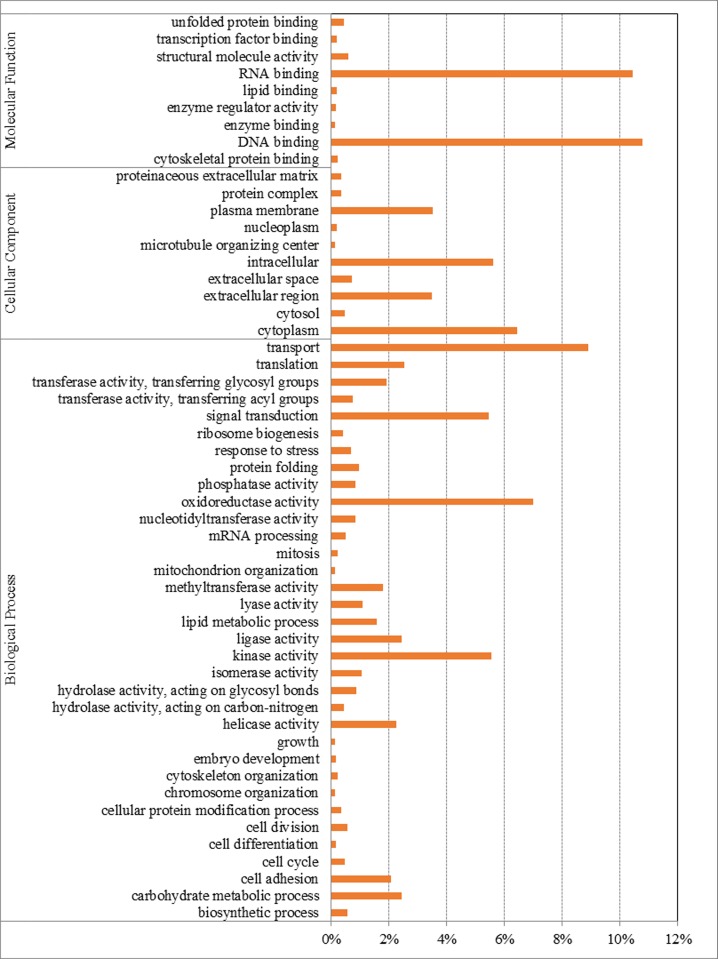
Gene Ontology (GO) analysis of *G*. *molesta* antennal transcripts. GO terms assigned to biological process, cellular component and molecular functions.

A total of 16,215 unigenes matched to known proteins in Genbank. Among the annotated unigenes, 64.3% had a first hits to Lepidopteran sequences. The top matched species were *Danaus plexippus* (52.2%), *Bombyx mori* (6.5%), *Tribolium castaneum* (4.4%), *Papilio xuthus* (4.1%), and *Acyrthosiphon pisum* (1.5%) (Fig 2). Fig 3 illustrated the distribution of the unigenes in GO terms. Among the 16,215 unigenes, 11,569 (71.35%) were assigned to 55,382 GO term annotations, with biological processes 26,297 terms; molecular function 15,062 terms; and cellular component 14,023 terms. In the biological process terms, transport, signal transduction and oxidoreductase activity were the mostly represented, while in the cellular component terms, the cytoplasm and intracellular were the most abundant. In the molecular function category, the genes expressed in the antennae were mostly enriched to DNA binding and RNA binding activity.

### Identification of putative odorant-binding proteins

**Fig 4:**
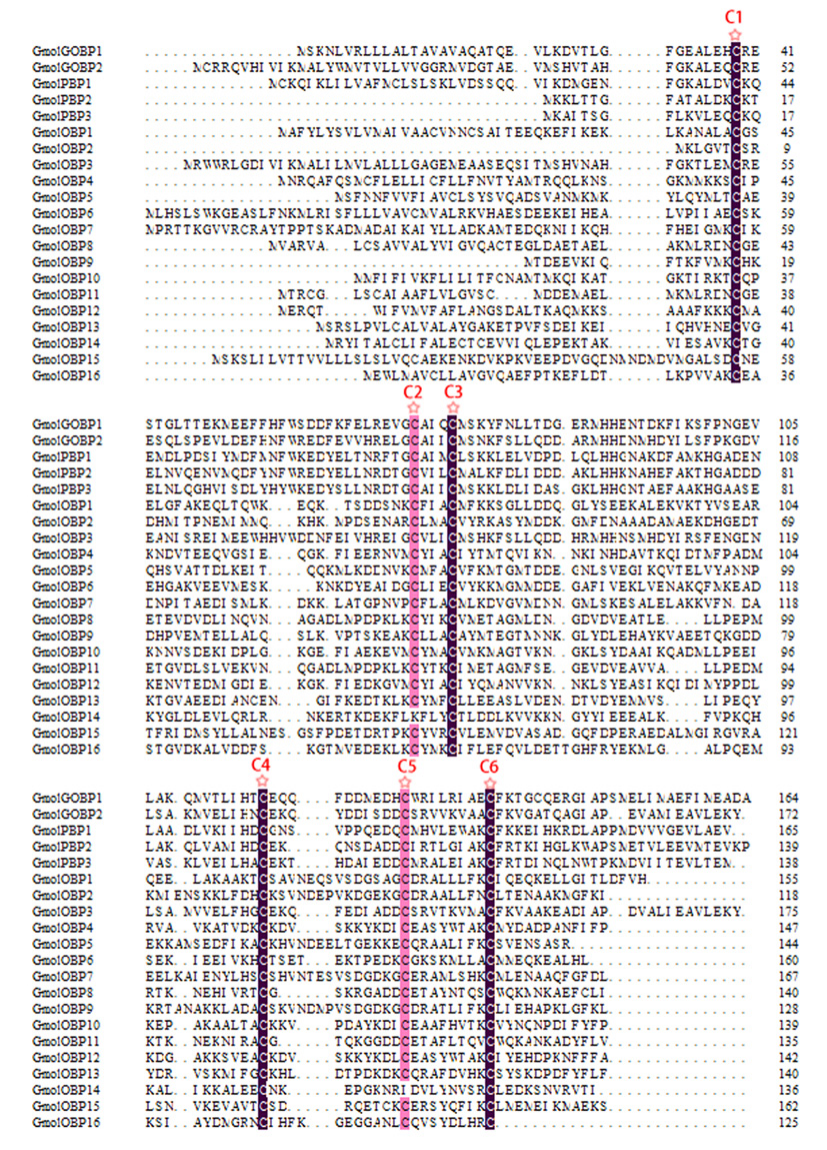
Sequences alignment of candidate GmolOBPs. The six conserved cysteine residues were marked with “☆”. As GmolOBP17, 18, 19, 20, 21, 22 and 23 are not intact sequences, those sequences are not included in the multisequence alignment.

**Fig 5:**
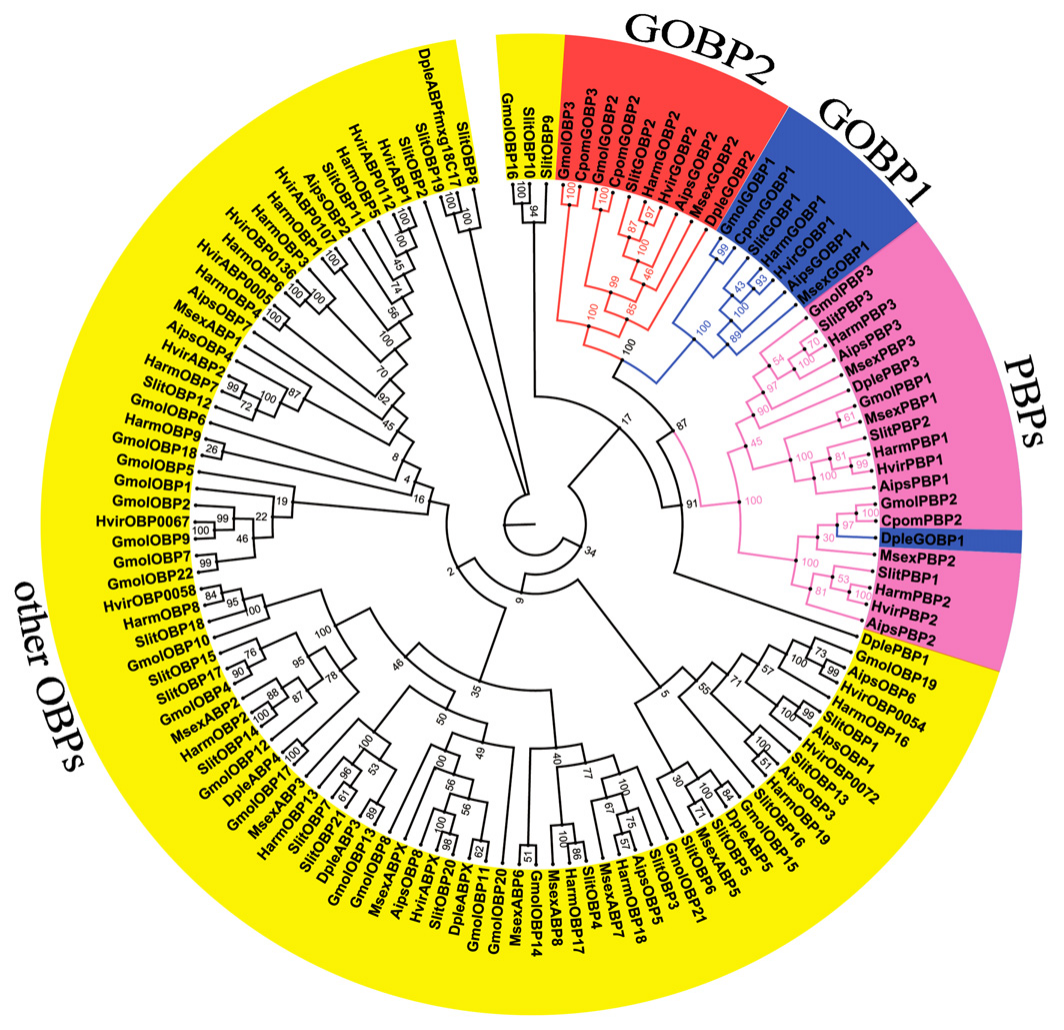
Neighbor-joining tree of candidate OBP genes from *G*. *molesta* and other Lepidoptera. The tree was drawn using Adobe Photoshop CS5, based on the unrooted tree constructed using BioNJ algorithm in Seaview v.4. The unrooted tree was constructed based on the sequence alignment obtained using MAFFT version 6. Gmol, *Grapholita molesta*, Cpom, *Cydia pomonella*, Dple, *Danaus plexippus*, Hvir, *Heliothis virescens*, Msex, *Manduca sexta*, Harm, *Helicoverpa armigera*, Aips, *Agrotis ipsilon*. The clade in blue indicates the GOBP1 gene clade; the clade in red indicates the GOBP2 clade, the clade in fuchsia indicates the PBPs clade, the yellow indicates other OBPs clade.

First, we used motif scanning to detect the conserved six cysteine residues pattern (C1-X20-66-C2-X3-C3-X21-43-C4-X8-14-C5-X8-C6 or C1-X15-39-C2-X3-C3-X21-44-C4-X7-12-C5-X8-C6, where X is any amino acid) of the candidate odorant-binding proteins [42]. We then used keyword searching and PSI-Blast. Twenty-eight sequences encoding putative odorant-binding proteins were identified, including two GOBPs and three PBPs. Of the 28 sequences, 21 had full ORFs, four unigenes had full-length ORFs, but without a signal peptide. Sequence alignment showed that almost all the putative OBPs shared the classic six-cysteine motif, except GmolOBP14, which was grouped into the “minus-C” subgroup with the second and fifth cysteine residues missing [43] (Fig 4). In the phylogenetic tree, as expected, the PBP and GOBP sequences were clustered into separate clades away from other OBPs. All the candidate OBP sequences with at least one lepidopteran ortholog were clustered in congruence with the BLAST results (Fig 5). Comparing our candidate OBPs with previously recorded OBPs of *G*.*molesta* in NCBI, 23 sequences as new genes, including GmolPBP1, GmolOBP1, GmolOBP2, and GmolOBP4 to GmolOBP23. The information on the OBPs is listed in Table 3. The nucleotide sequences are listed in S2 File.

**Table 3:** Unigenes of candidate odorant binding proteins.

Unigene reference	Gene name	Length (nt)	ORF (aa)	BLASTx best hit (Reference/Name/Species)	E-value	Identify	Full length	Signal Peptide
**Pheromone binding protein**								
Comp42972_c0_seq8	GmolPBP1	2123	166	gb|AAF06142.1|pheromone binding protein [Synanthedon exitiosa]	9e-62	63%	Yes	Yes
Comp43708_c4_seq3	GmolPBP2	2409	141	gb|AFL91693.1|pheromone binding protein 2 [Cydia pomonella]	1e-96	97%	Yes	No
comp35908_c0_seq2	GmolPBP3	665	139	gb|AFD34183.1|pheromone binding protein 2 [Argyresthia conjugella]	1e-100	100%	Yes	Yes
**General odorant binding protein**								
Comp43859_c0_seq4	GmolGOBP1	2512	165	gb|AFH02841.1|general odorant binding protein 1 [Grapholita molesta]	7e-90	99%	Yes	Yes
comp35716_c0_seq1	GmolGOBP2	1063	173	gb|AFH02842.1|general odorant binding protein 2 [Grapholita molesta]	5e-111	99%	Yes	Yes
**Other odorant binding protein**								
comp34669_c0_seq1	GmolOBP1	980	156	gb|AFD34177.1|odorant binding protein 1 [Argyresthia conjugella]	4e-26	42%	Yes	Yes
comp32473_c0_seq5	GmolOBP2	505	119	ref|NP_001140188.1|odorant-binding protein 4 [Bombyx mori]	2e-39	52%	Yes	No
comp38984_c0_seq2	GmolOBP3	2608	176	gb|AFP66959.1|general odorant binding protein 3 [Cydia pomonella]	2e-90	99%	Yes	Yes
comp21044_c0_seq1	GmolOBP4	798	148	gb|AAL60415.1|AF393490_1antennal binding protein 4 [Manduca sexta]	7e-66	75%	Yes	Yes
comp35668_c0_seq1	GmolOBP5	588	145	ref|NP_001140189.1|odorant-binding protein 5 precursor [Bombyx mori]	3e-28	41%	Yes	Yes
comp35533_c1_seq1	GmolOBP6	849	161	gb|AGH70102.1|odorant binding protein 6 [Spodoptera exigua]	9e-20	36%	Yes	Yes
comp35668_c0_seq2	GmolOBP7	906	168	gb|EHJ67765.1|odorant binding protein [Danaus plexippus]	7e-55	63%	Yes	No
comp37376_c1_seq1	GmolOBP8	832	141	gb|AFD34174.1|antennal binding protein X [Argyresthia conjugella]	3e-49	62%	Yes	Yes
Comp39973_c0_seq2	GmolOBP9	631	129	gb|AEX07279.1|odorant-binding protein [Helicoverpa armigera]	9e-54	64%	Yes	No
comp40035_c5_seq2	GmolOBP10	1799	140	gb|AFG73000.1|odorant-binding protein 2 [Cnaphalocrocis medinalis]	3e-65	72%	Yes	Yes
comp41846_c1_seq1	GmolOBP11	788	136	gb|AGK24582.1|antennal-binding protein X [Chilo suppressalis]	5e-63	78%	Yes	Yes
Comp43336_c6_seq1	GmolOBP12	862	143	gb|AEB54586.1|OBP2 [Helicoverpa armigera]	2e-60	64%	Yes	Yes
comp26714_c0_seq1	GmolOBP13	1387	141	gb|AFD34173.1|odorant binding protein 5 [Argyresthia conjugella]	9e-74	77%	Yes	Yes
comp28072_c0_seq1	GmolOBP14	548	137	gb|AGK24577.1|odorant-binding protein 1 [Chilo suppressalis]	3e-11	29%	Yes	Yes
comp36383_c0_seq1	GmolOBP15	1331	163	gb|AGI37367.1|pheromone binding protein 3 [Cnaphalocrocis medinalis]	3e-39	51%	Yes	Yes
comp39806_c0_seq1	GmolOBP16	1232	242	gb|AGH70107.1|odorant binding protein 11 [Spodoptera exigua]	8e-63	71%	Yes	Yes
comp41079_c0_seq11	GmolOBP17	1094	96	gb|AFG72998.1|odorant-binding protein 1 [Cnaphalocrocis medinalis]	8e-63	71%	No	Yes
comp61252_c0_seq1	GmolOBP18	250	76	gb|EHJ64212.1|odorant-binding protein 2 [Danaus plexippus]	2e-34	76%	No	Yes
comp2648_c0_seq1	GmolOBP19	421	94	gb|AGK24580.1|odorant-binding protein 4 [Chilo suppressalis]	2e-52	80%	No	No
comp31482_c0_seq1	GmolOBP20	422	124	gb|AFD34182.1|odorant binding protein 6 [Argyresthia conjugella]	5e-54	66%	No	Yes
comp33414_c0_seq1	GmolOBP21	324	95	gb|EHJ66992.1|antennal binding protein [Danaus plexippus]	1e-27	61%	No	Yes
comp33722_c0_seq2	GmolOBP22	373	123	ref|NP_001153664.1|odorant binding protein [Bombyx mori]	2e-22	44%	No	No
comp41079_c0_seq1	GmolOBP23	1640	75	gb|EHJ65654.1|antennal binding protein 4 [Danaus plexippus]	9e-12	61%	No	No

### Identification of candidate chemosensory proteins

**Fig 6:**
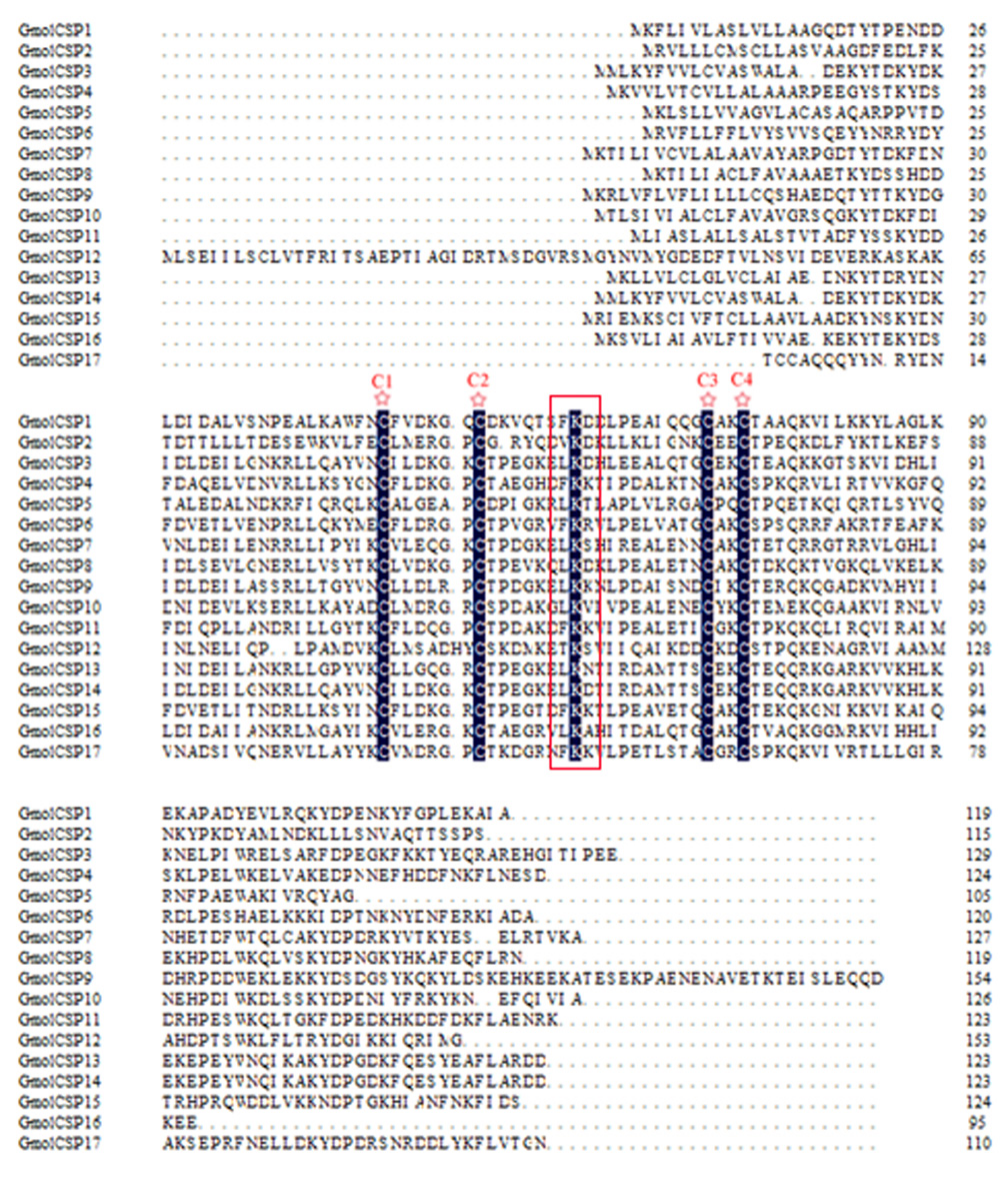
Sequences alignment of candidate GmolCSPs. The four conserved cysteine residues were marked with “☆”, a conserved lysine was indicated with a box.

**Fig 7:**
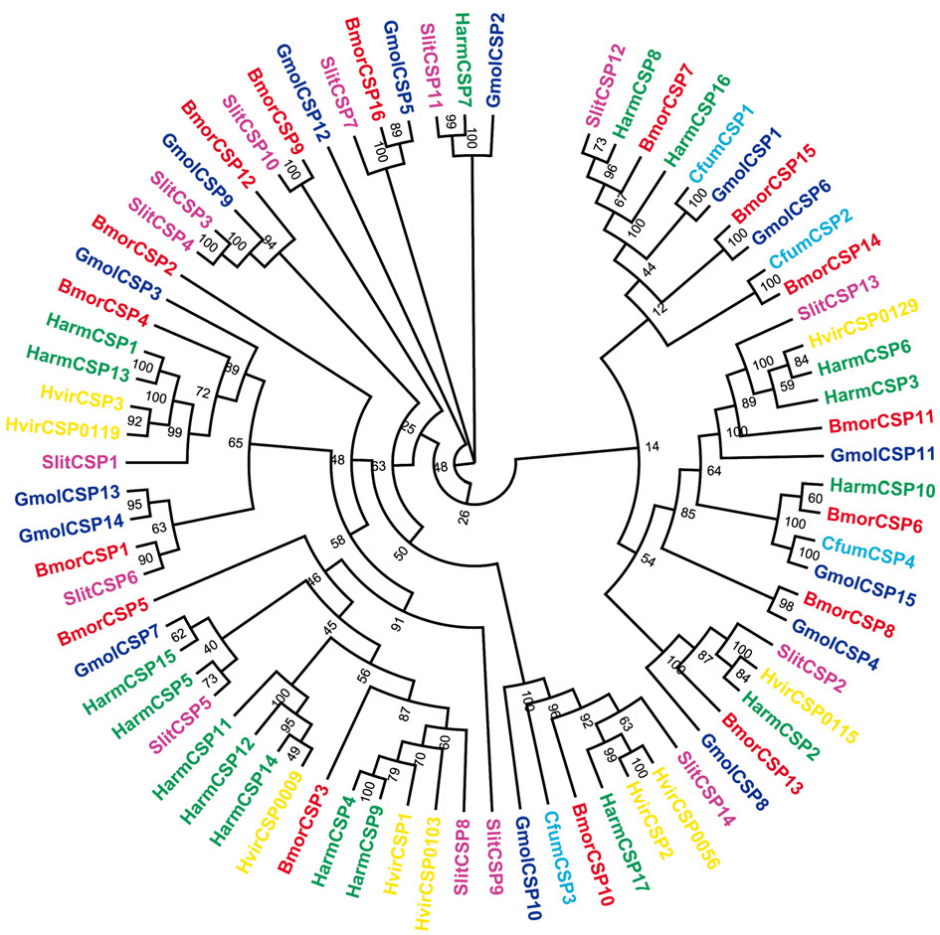
Neighbor-joining tree of candidate CSP genes from *G*. *molesta* and other Lepidoptera. The tree was drawn with Adobe Photoshop CS5, based on the unrooted tree constructed using the BioNJ algorithm in Seaview v.4. The unrooted tree was constructed based on the sequence alignment produced using MAFFT version 6. Gmol, *Grapholita molesta*, Bmor, *Bombyx mori*, Cfum, *Choristioneure fumiferana*, Harm, *Helicoverpa armigera*, Hvir, *Heliothis virescens*, Slit, *Spodoptera Iittoralis*. Color of gene names indicates species.

Seventeen different sequences encoding putative chemosensory proteins were identified within the *G*. *molesta* antennal transcriptome. Sequence analysis identified 15 unigenes with full-length ORFs and 16 unigenes with predicted signal peptide. One unigene without signal peptide since truncate at the 5'-end. The four conserved cysteine (with a pattern of C1-X6-8-C2-X18-19-C3-X2-C4) were found in all the 17 candidate GmolCSPs [44] (Fig 6). In addition to the conserved cysteine residues, a lysine located between the second and third cysteine was also conserved in all sequences. Neighbor-joining tree analysis showed that all of the 17 sequences were clustered with Lepidopteran orthologous genes (Fig 7). These candidate CSPs were named as “GmolCSP” followed by a numeral. The information on the CSPs is listed in Table 4. The sequences are listed in S2 File.

**Table 4:** Unigenes of candidate chemosensory proteins.

Unigene reference	Gene name	Length (nt)	ORF (aa)	BLASTx best hit (Reference/Name/Species)	E-value	Identify	Full length	Signal Peptide
comp29970_c0_seq1	GmolCSP1	384	120	gb|AAR84077.1|chemosensory protein 1, partial [Choristoneura fumiferana]	2e-44	69%	Yes	Yes
comp30965_c0_seq1	GmolCSP2	689	116	gb|AGI37361.1|chemosensory protein 1 [Cnaphalocrocis medinalis]	1e-06	36%	Yes	Yes
comp38507_c0_seq3	GmolCSP3	1506	130	gb|AAK53762.1|AF368375_1chemosensory protein [Helicoverpa armigera]	1e-47	70%	Yes	Yes
comp31529_c0_seq2	GmolCSP4	520	125	gb|EHJ70186.1|chemosensory protein [Danaus plexippus]	6e-46	70%	Yes	Yes
comp33568_c0_seq1	GmolCSP5	427	106	ref|NP_001091782.1|chemosensory protein 16 precursor [Bombyx mori]	2e-52	88%	Yes	Yes
comp39117_c0_seq1-1	GmolCSP6	970	121	gb|AAW23971.1|chemosensory protein 4 [Choristoneura fumiferana]	2e-70	88%	Yes	Yes
comp32406_c0_seq1	GmolCSP7	527	128	gb|ABM67687.1|chemosensory protein CSP2 [Plutella xylostella]	4e-54	73%	Yes	Yes
comp35615_c0_seq1	GmolCSP8	956	120	gb|AEX07265.1|CSP2 [Helicoverpa armigera]	3e-43	60%	Yes	Yes
comp37677_c0_seq3	GmolCSP9	1441	156	dbj|BAM18557.1|protein serine/threonine kinase [Papilio xuthus]	7e-48	71%	Yes	Yes
comp41217_c1_seq3	GmolCSP10	1262	127	gb|AFQ32775.1|chemosensory protein [Grapholita molesta]	2e-48	60%	Yes	Yes
comp41050_c0_seq1	GmolCSP11	681	124	emb|CAJ01505.1|hypothetical protein [Manduca sexta]	1e-55	80%	Yes	Yes
comp40028_c0_seq2	GmolCSP12	1396	154	gb|EHJ76400.1|hypothetical protein KGM_11196 [Danaus plexippus]	6e-44	60%	Yes	Yes
comp38507_c0_seq4	GmolCSP13	828	124	ref|NP_001037065.1|chemosensory protein 1 [Bombyx mori]	7e-42	63%	Yes	Yes
comp38507_c0_seq2	GmolCSP14	830	124	dbj|BAF91711.1|chemosensory protein [Papilio xuthus]	1e-40	61%	Yes	Yes
comp39117_c0_seq1-2	GmolCSP15	970	125	gb|AAW23971.1|chemosensory protein 4 [Choristoneura fumiferana]	2e-70	88%	Yes	Yes
comp20508_c0_seq1	GmolCSP16	308	95	ref|NP_001037062.1|chemosensory protein 5 precursor [Bombyx mori]	2e-28	53%	No	Yes
comp26710_c0_seq1	GmolCSP17	358	76	gb|AAR84078.1|chemosensory protein 2 [Choristoneura fumiferana]	1e-66	91%	No	No

### Identification of candidate odorant receptors

**Fig 8:**
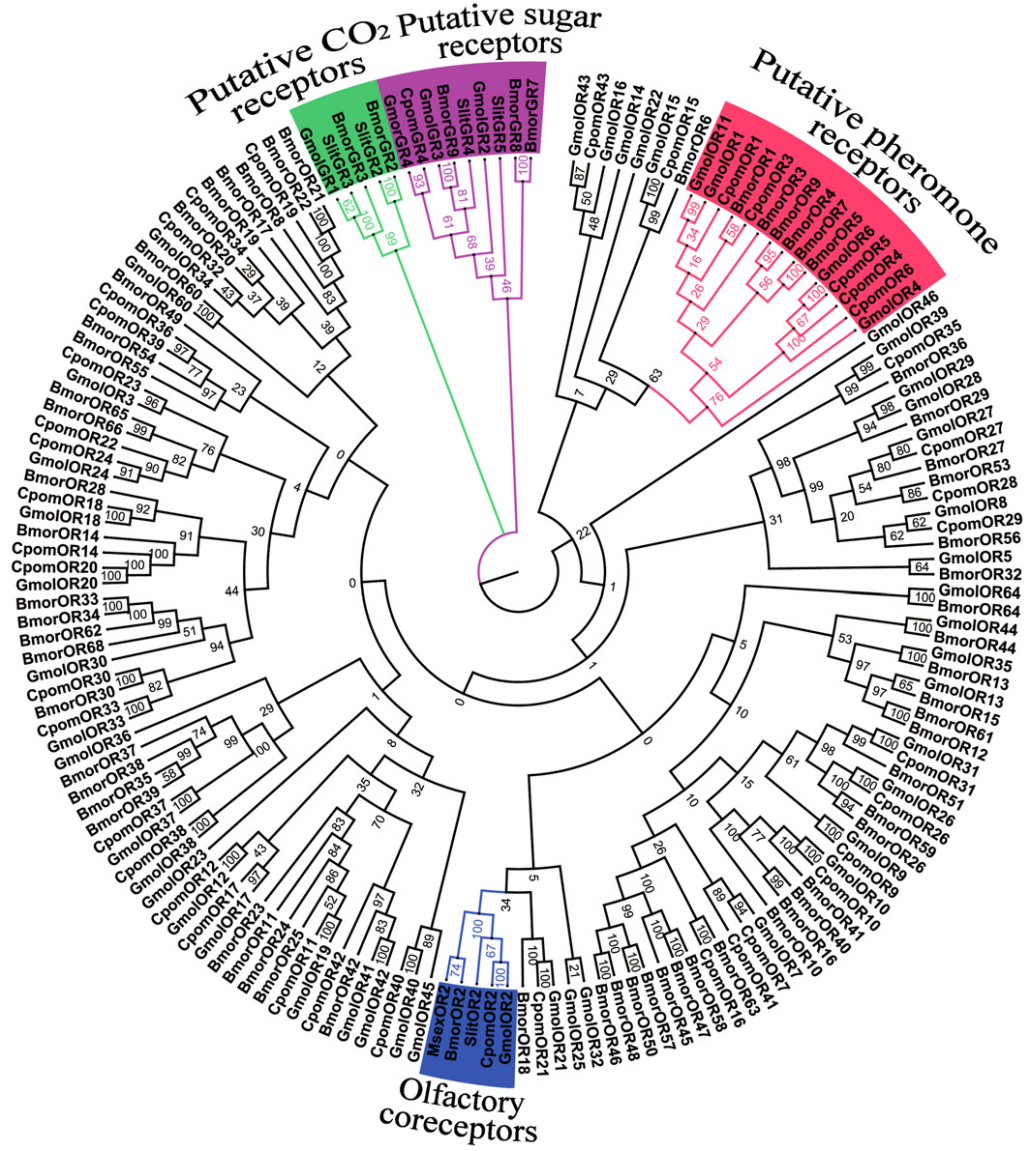
Neighbor-joining tree of candidate odorant receptor (OR) and gustatory receptor (GR) from *G*. *molesta* and other Lepidoptera. The tree was drawn using Adobe Photoshop CS5, based on an unrooted tree constructed using the BioNJ algorithm in Seaview v.4. The unrooted tree was constructed based on the sequence alignment produced using MAFFT version 6. Gmol, *Grapholita molesta*, Msex, *Manduca sexta*, Cpom, *Cydia pomonella*, Slit, *Spodoptera Iittoralis*, Bmor, *Bombyx mori*.

Bioinformatic analysis identified 48 different sequences encoding putative ORs and four sequences encoding putative GRs. GmolORs and GmolGRs were named according to their similarities with previously annotated Lepidoptera ORs and the topology predictions from TMpred as observed from other insect ORs [45]. Twelve of these sequences appeared to contain full lengths genes since they had full length ORFs with 5–8 transmembrane domains (Table 5). The co-receptor of *G*.*molesta* showed 95% identity to *C*. *pomonella* co-receptor, CpomOR2, one of the most conserved co-receptors in insect species. While similar with other insect ORs, most GmolORs were highly divergent and shared low similarity with other insect ORs, except for closely related species such as *Cydia pomonella*. Four candidate GmolORs sequences (GmolOR1, GmolOR4, GmolOR6, and GmolOR11) tended to be pheromone receptors (PRs) as they are highly conserved with CpomOR fragments and BmorPRs amino acid sequences from other species [46]. These four sequences were clusterd into one subgroup in the phylogenetic tree (Fig 8). The gustatory receptors that we identified (GmolGR2, GmolGR3, and GmolGR4) were found in a clade with sugar receptors, which included gustatory receptors identified from other moth antennae; these gustatory receptors were also clustered in this clade (Fig 8) [39,47,48]. Another putative GR, named GmolGR1, was clustered with putative CO_2_ receptors. The information of ORs and GRs are given in Table 5, the nucleotide sequences are listed in S2 File.

**Table 5:** Unigenes of candidate ordoart receptors and gustatory receptors.

Unigene reference	Gene name	Length (nt)	ORF (aa)	BLASTx best hit (Reference/Name/Species)	E-value	Identify	Full length	TMD (No)
**Co-receptor**								
comp42201_c0_seq1	GmolOR2	2730	473	gb|AFC91712.1| putative odorant receptor OR2 [Cydia pomonella]	0.0	95%	Yes	7
**Pheromone receptors**								
comp44030_c0_seq5	GmolOR1	884	88	gb|AFC91711.1| putative odorant receptor OR1, partial [Cydia pomonella]	3e-29	52%	No	1
comp37031_c0_seq3	GmolOR4	1624	430	gb|AGG91650.1| odorant receptor [Ostrinia furnacalis]	6e-103	40%	No	5
comp52214_c0_seq1	GmolOR6	377	118	gb|AFC91716.1| putative odorant receptor OR6, partial [Cydia pomonella]	2e-53	70%	No	0
comp36128_c0_seq1	GmolOR11	536	135	gb|AFK30397.1| odorant receptor 4 [Ostrinia furnacalis]	4e-36	53%	No	3
**Other odorant receptors**								
comp39216_c0_seq2	GmolOR3	1400	420	gb|EHJ75140.1| olfactory receptor [Danaus plexippus]	5e-65	70%	Yes	6
comp40794_c1_seq1	GmolOR5	1980	414	dbj|BAH66328.1| olfactory receptor [Bombyx mori]	3e-119	51%	No	6
comp42525_c0_seq1	GmolOR7	1213	395	gb|AFC91717.1| putative odorant receptor OR7, partial [Cydia pomonella]	3e-124	86%	Yes	8
comp37543_c0_seq4	GmolOR8	615	199	ref|NP_001166617.1| olfactory receptor 56 [Bombyx mori]	2e-82	59%	No	2
comp36812_c0_seq5	GmolOR9	1707	395	gb|AFC91718.1| putative odorant receptor OR9, partial [Cydia pomonella]	3e-99	94%	Yes	5
comp43031_c0_seq3	GmolOR10	2363	397	gb|AFC91719.1| putative odorant receptor OR10 [Cydia pomonella]	0.0	84%	Yes	7
comp40338_c0_seq2	GmolOR12	1224	399	gb|AFC91721.1| putative odorant receptor OR12 [Cydia pomonella]	0.0	90%	Yes	7
comp42372_c0_seq4	GmolOR13	958	210	tpg|DAA05974.1| TPA_exp: odorant receptor 15 [Bombyx mori]	6e-74	53%	No	4
comp36822_c0_seq2	GmolOR14	439	136	gb|EHJ67735.1| olfactory receptor [Danaus plexippus]	1e-04	28%	No	2
comp41698_c0_seq2	GmolOR15	946	227	gb|AFC91723.1| putative odorant receptor OR15 [Cydia pomonella]	5e-159	89%	No	1
comp42516_c0_seq34	GmolOR16	1458	177	gb|AFC91724.1| putative odorant receptor OR16 [Cydia pomonella]	6e-89	77%	No	0
comp30377_c0_seq2	GmolOR17	953	254	gb|AFC91725.1| putative odorant receptor OR17 [Cydia pomonella]	7e-174	84%	No	4
comp40766_c0_seq4	GmolOR18	998	238	gb|AFC91726.1| putative odorant receptor OR18 [Cydia pomonella]	0.0	90%	No	3
comp42164_c0_seq3	GmolOR19	1274	398	emb|CAG38113.1| putative chemosensory receptor 12 [Heliothis virescens]	3e-12	43%	No	4
comp43037_c0_seq1	GmolOR20	1501	427	gb|AFC91728.1| putative odorant receptor OR20 [Cydia pomonella]	0.0	87%	Yes	7
comp41733_c0_seq1	GmolOR21	1334	376	gb|AFC91729.1| putative odorant receptor OR21 [Cydia pomonella]	0.0	90%	Yes	5
comp36128_c0_seq3	GmolOR22	842	279	gi|205361596|dbj|BAG71417.1| olfactory receptor-1 [Diaphania indica]	3e-40	32%	No	2
comp20621_c0_seq1	GmolOR23	419	139	emb|CAD31850.1| putative chemosensory receptor 1 [Heliothis virescens]	6e-19	45%	No	0
comp42767_c0_seq45	GmolOR24	1716	259	gb|AFC91732.1| putative odorant receptor OR24 [Cydia pomonella]	8e-163	74%	No	3
comp33674_c0_seq3	GmolOR25	1054	279	gb|EHJ78030.1| olfactory receptor 29 [Danaus plexippus]	4e-155	75%	No	0
comp41262_c0_seq4	GmolOR26	1462	315	gb|AFC91734.1| putative odorant receptor OR26 [Cydia pomonella]	6e-128	87%	No	5
comp39775_c0_seq1	GmolOR27	1281	402	ref|NP_001166893.1| olfactory receptor 27 [Bombyx mori]	1e-152	64%	No	5
comp39835_c0_seq3	GmolOR28	1686	398	ref|NP_001166894.1| olfactory receptor 29 [Bombyx mori]	2e-142	49%	No	5
comp41754_c3_seq2	GmolOR29	738	238	tpg|DAA05985.1| TPA_exp: odorant receptor 29 [Bombyx mori]	1e-95	59%	No	3
comp36822_c0_seq1	GmolOR30	1081	181	gb|AFC91738.1| putative odorant receptor OR30 [Cydia pomonella]	1e-28	40%	No	0
comp44215_c1_seq7	GmolOR31	1380	402	gb|AFC91739.1| putative odorant receptor OR31 [Cydia pomonella]	0.0	79%	No	4
comp43530_c1_seq3	GmolOR32	1420	444	ref|NP_001116817.1| olfactory receptor-like [Bombyx mori]	3e-174	58%	Yes	6
comp41607_c0_seq3	GmolOR33	2364	374	gb|AFC91741.1| putative odorant receptor OR33 [Cydia pomonella]	4e-141	76%	Yes	6
comp38817_c0_seq3	GmolOR34	1415	424	gb|AFC91742.1| putative odorant receptor OR34 [Cydia pomonella]	3e-102	43%	No	3
comp41935_c2_seq6	GmolOR35	1198	334	ref|XP_004067596.1| PREDICTED: protein LOC101171734 [Oryzias latipes]	3e-113	55%	Yes	6
comp40388_c2_seq1	GmolOR36	1277	355	gb|AET06162.1| odorant receptor 3, partial [Planotortrix notophaea]	0.0	82%	No	6
comp44364_c1_seq2	GmolOR37	1938	398	gb|AFC91745.1| putative odorant receptor OR37 [Cydia pomonella]	3e-173	82%	No	4
comp44203_c1_seq3	GmolOR38	2158	437	gb|AFC91746.1| putative odorant receptor OR38 [Cydia pomonella]	0.0	65%	No	5
comp40263_c1_seq4	GmolOR39	1403	414	ref|NP_001166892.1| olfactory receptor 36 [Bombyx mori]	2e-127	48%	No	4
comp43824_c0_seq2	GmolOR40	1841	394	gb|AFC91748.1| putative odorant receptor OR40 [Cydia pomonella]	3e-123	71%	No	4
comp38057_c0_seq1	GmolOR41	462	108	ref|NP_001091818.1| olfactory receptor 42 [Bombyx mori]	7e-39	60%	No	1
comp38057_c0_seq2	GmolOR42	773	252	gb|AFC91750.1| putative odorant receptor OR42 [Cydia pomonella]	7e-123	83%	No	4
comp33512_c0_seq2	GmolOR43	741	90	gb|AFC91751.1| putative odorant receptor OR43 [Cydia pomonella]	1e-09	57%	No	0
comp34069_c0_seq3	GmolOR44	1532	436	gb|AGG08877.1| putative olfactory receptor 44 [Spodoptera litura]	0.0	72%	No	6
comp41837_c0_seq1	GmolOR45	1455	401	emb|CBW30700.1| odorant receptor [Drosophila simulans]	3e-23	26%	No	5
comp36742_c0_seq1	GmolOR46	1739	495	dbj|BAM19586.1| similar to CG13607, partial [Papilio xuthus]	0.0	80%	No	5
comp41612_c0_seq4	GmolOR60	1647	450	ref|NP_001155301.1| olfactory receptor 60 [Bombyx mori]	0.0	70%	Yes	6
comp39091_c0_seq2	GmolOR64	1298	416	ref|NP_001166621.1| olfactory receptor 64 [Bombyx mori]	9e-97	53%	No	6
**Gustatory receptors**								
comp29992_c0_seq1	GmolGR1	1047	331	gb|EHJ78216.1|gustatory receptor 24 [Danaus plexippus]	3e-138	77%	No	5
comp32055_c0_seq3	GmolGR2	407	96	tpg|DAA06387.1|TPA_inf: gustatory receptor 50 [Bombyx mori]	1e-33	66%	No	2
comp39217_c0_seq1	GmolGR3	1186	258	gb|AGA04648.1|gustatory receptor [Helicoverpa armigera]	3e-152	76%	No	3
comp25108_c0_seq3	GmolGR4	706	136	gb|AFC91733.1| putative odorant receptor OR25 [Cydia pomonella]	2e-36	46%	No	2

### Identification of candidate ionotropic receptors

**Fig 9:**
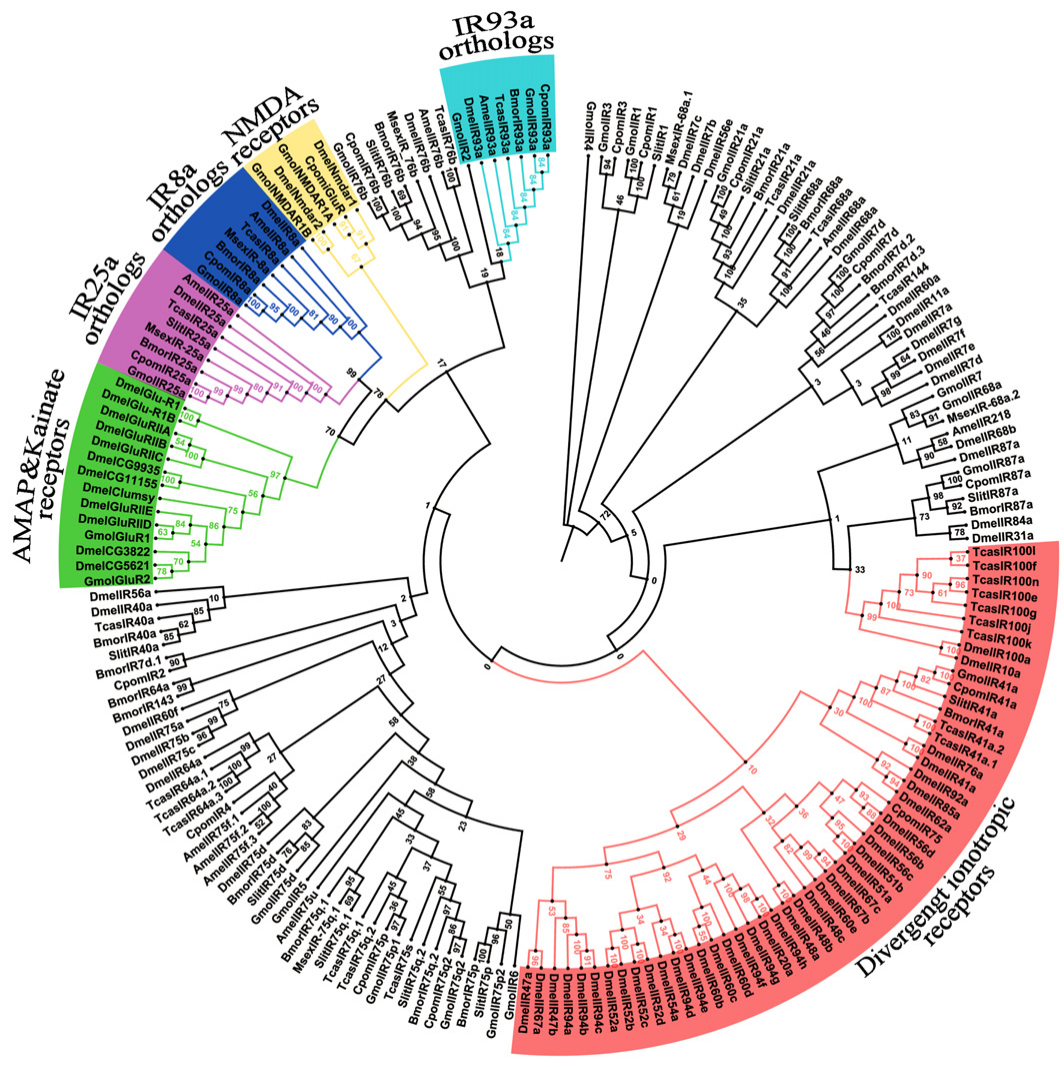
Neighbor-joining tree of candidate ionotropic receptor (IR) genes from *G*. *molesta* and other insects. The tree was drawn using Adobe Photoshop CS5, based on an unrooted tree constructed using the BioNJ algorithm in Seaview v.4. The unrooted tree was constructed based on a sequence alignment produced using MAFFT version 6. Gmol, *Grapholita molesta*, Msex, *Manduca sexta*, Cpom, *Cydia pomonella*, Slit, *Spodoptera Iittoralis*, Bmor, *Bombyx mori*, Dmel, *Drosophila melanogaster*, Amel, *Apis mellifera*, Tcas, *Tribolium castaneum*.

Twenty four sequences encoding putative IRs proteins were also identified in the *G*. *molesta* antennal transcriptome. The alignment revealed that all the 24 sequences represent unique genes since they possessed overlapping regions without identity. Ten of the IRs appeared to contain a full length ORFs (GmolIR8a, 25a, 21a, 75p2, 76b, 87a, 93a, 7d, 5 and NMDAR1B), and were longer than 1700 bp in general. In addition, it was predicted that three transmembrane domains existed in all 10 sequences by TMHMM 2.0, the typical characteristic of IRs. The remaining 14 sequences were truncate at either 5' or 3' terminus. Neighbor-joining tree analysis revealed that all putative IRs were found to have orthologoues from *B*. *mori*, *M*. *sexta*, *C*. *pomonella*, *S*. *littoralis*, *D*. *melanogaster*, *A*. *mellifera*, and *T*. *castaneum*. According to their positions in the phylogenetic tree and the strong bootstrap support, 15 of 24 putative *G*. *molesta* IRs were given names that are consistent with the number and suffix of the Dmel/Bmor/Slit/Cpom/Amel/Tcas IR orthologues in the same clade. Two of the remaining nine IR sequences, comp37980_c0_seq2 and comp40732_c0_seq6, clustered with their ionotropic receptor orthologues into N-methyl-D-aspartic acid receptor (NMDA receptors) clade, and these were named “GmolNMDAR1A” and “GmolNMDAR1B”. The other two sequences, comp5756_c0_seq1 and comp5467_c0_seq1, clustered with their ionotropic receptor orthologues of α-Amino-3-hydroxy-5-methylisoxazole-4-propionic acid (AMAP) and Kainate receptors clade, and these were named “GmolGluIR1” and “GmolGluIR2”, respectively. The remaining five unigenes, comp52557_c0_seq1, comp36336_c0_seq3, comp41705_c0_seq15, comp43295_c0_seq6, and comp43552_c0_seq13, did not show meanful similarity with known IR encoding genes but with conserved structural features, and thus were named as “GmolIR2”, “GmolIR4”, “GmolIR5”, “GmolIR6” and “GmolIR7”, respectively (Fig 9). The information including the unigene reference, length, and first BLASTX hit of all the 24 IRs are given in Table 6. The sequences of all the 24 IRs are listed in S2 File.

**Table 6:** Unigenes of candidate ionotropic receptors.

Unigene reference	Gene name	Length (nt)	ORF (aa)	BLASTx best hit (Reference/Name/Species)	E-value	Identify	Full length	TMD (No)
comp44508_c0_seq1	GmolIR8a	2971	892	gb|AFC91764.1|putative ionotropic receptor IR8a, partial [Cydia pomonella]	2e-22	54%	Yes	3
comp43733_c0_seq1	GmolIR25a	3490	923	gb|AFC91757.1|putative ionotropic receptor IR25a [Cydia pomonella]	0.0	96%	Yes	3
comp42937_c0_seq1	GmolIR21a	2722	853	gb|AFC91761.1|putative ionotropic receptor IR21a, partial [Cydia pomonella]	0.0	88%	Yes	3
comp41847_c1_seq3	GmolIR41a	1846	536	gb|AFC91758.1|putative ionotropic receptor IR41a [Cydia pomonella]	0.0	91%	No	2
comp34810_c0_seq9	GmolIR68a	1425	411	gb|ADR64682.1|putative chemosensory ionotropic receptor IR68a [Spodoptera littoralis]	4e-173	66%	No	0
comp43328_c0_seq3	GmolIR75d	1225	380	gb|ADR64683.1|putative chemosensory ionotropic receptor IR75d [Spodoptera littoralis]	6e-105	50%	No	1
comp43295_c0_seq14	GmolIR75p1	523	174	gb|AFC91755.1|putative ionotropic receptor IR75p, partial [Cydia pomonella]	5e-119	95%	No	0
comp43826_c0_seq19	GmolIR75p2	1958	605	gb|ADR64684.1|putative chemosensory ionotropic receptor IR75p [Spodoptera littoralis]	2e-143	43%	Yes	3
comp43424_c0_seq31	GmolIR75q2	2528	313	gb|AFC91752.1|putative ionotropic receptor IR75q2 [Cydia pomonella]	0.0	89%	No	1
comp41154_c0_seq4	GmolIR76b	3326	440	gb|AFC91765.1|putative ionotropic receptor IR76b [Cydia pomonella]	0.o	84%	Yes	3
comp44120_c0_seq14	GmolIR87a	1734	513	gb|AFC91760.1|putative ionotropic glutamate receptor 87a, partial [Cydia pomonella]	0.0	88%	Yes	3
comp44415_c0_seq2	GmolIR93a	2713	870	gb|AFC91753.1|putative ionotropic receptor IR93a, partial [Cydia pomonella]	0.0	92%	Yes	4
comp37980_c0_seq2	GmolNMDAR1A	1626	361	gb|EHJ78211.1|putative NMDA-type glutamate receptor 1 [Danaus plexippus]	0.0	96%	No	2
comp40732_c0_seq6	GmolNMDAR1B	1780	309	ref|NP_001040129.2|glutamate [NMDA] receptor-associated protein 1 [Bombyx mori]	5e-108	78%	Yes	3
comp5756_c0_seq1	GmolGluR1	339	113	gb|EGI61384.1|Glutamate receptor, ionotropic kainate 1 [Acromyrmex echinatior]	1e-64	91%	No	2
comp5467_c0_seq1	GmolGluIR2	358	118	ref|XP_001655460.1|ionotropic glutamate receptor subunit ia [Aedes aegypti]	2e-60	81%	No	1
comp42373_c0_seq9	GmolIR1	1444	458	gb|AFC91754.1|putative ionotropic receptor IR1, partial [Cydia pomonella]	2e-154	73%	No	2
comp43818_c0_seq8	GmolIR3	1042	138	gb|AFC91767.1|putative ionotropic receptor IR3, partial [Cydia pomonella]	2e-41	69%	No	0
comp27491_c0_seq3	GmolIR7d	1854	549	gb|AFC91766.1|putative ionotropic receptor IR7d, partial [Cydia pomonella]	1e-113	87%	Yes	4
comp52557_c0_seq1	GmolIR2	401	133	gb|EHJ63562.1|metabotropic glutamate receptor B [Danaus plexippus]	7e-87	97%	No	2
comp36336_c0_seq3	GmolIR4	560	186	gb|EHJ74994.1|putative ionotropic glutamate receptor-invertebrate [Danaus plexippus]	3e-10	55%	No	0
comp41705_c0_seq15	GmolIR5	2529	579	ref|XP_001651553.1|ionotropic glutamate receptor-invertebrate [Aedes aegypti]	1e-75	32%	Yes	3
comp43295_c0_seq6	GmolIR6	996	331	gb|EHJ72019.1|putative ionotropic glutamate receptor-invertebrate [Danaus plexippus]	2e-104	74%	No	1
comp43552_c0_seq13	GmolIR7	985	239	gb|ADM88008.1|ionotropic GABA-aminobutyric acid receptor RDL1-3b6a [Bombyx mori]	2e-142	96%	No	0

### Identification of candidate sensory neuron membrane proteins

Both SNMP1 and SNMP2 were obtained from *G*. *molesta* antennal transcriptome. In comparison, GmolSNMP1 has 68% identity with SNMP1 of *Plutella xylostella* (GenBank accession number: E2IHA6.1), while GmolSNMP2 has 69% identity with SNMP2 of *Ostrinia nubilalis* (GenBank accession number: E5EZW9.1). GmolSNMP1 had a full ORF with two transmembrane domains at N-terminus and C-terminus, respectively, while GmolSNMP2 was incomplete due to truncation at the 3' terminus. In addition, we also identified an odor degrading enzyme gene (ODE). ODE is responsible in the signal inactivation step and it rapidly degrades the stimulatory odorant molecules. The information including the unigene reference, length, and best BLASTX hit of SNMPs and ODE was listed in Table 7. The sequences of two SNMPs and an ODE were listed in S2 File.

**Table 7:** Unigenes of candidate sensory neuron membrane proteins and odor degrading enzyme.

Unigene reference	Gene name	Length (nt)	ORF (aa)	BLASTx best hit (Reference/Name/Species)	E-value	Identify	Full length	TMD (No)
**Sensory neuron membrane proteins**								
comp44414_c0_seq1	GmolSNMP1	1816	489	gnl|BL_ORD_ID|1375793 sensory neuron membrane protein-1 [Plutella xylostella]	0.0	68%	Yes	2
comp36266_c0_seq1	GmolSNMP2	1868	518	gnl|BL_ORD_ID|1536434 sensory neuron membrane protein 2 [Ostrinia nubilalis]	0.0	69%	No	1
**Odor degrading enzyme**								
comp41848_c0_seq1	GmolODE	1923	540	gb|AAM14415.1|putative odorant-degrading enzyme [Antheraea polyphemus]	0.0	65%	yes	

### Tissue and sex-specific expression of candidate OBP and CSP genes

**Fig 10:**
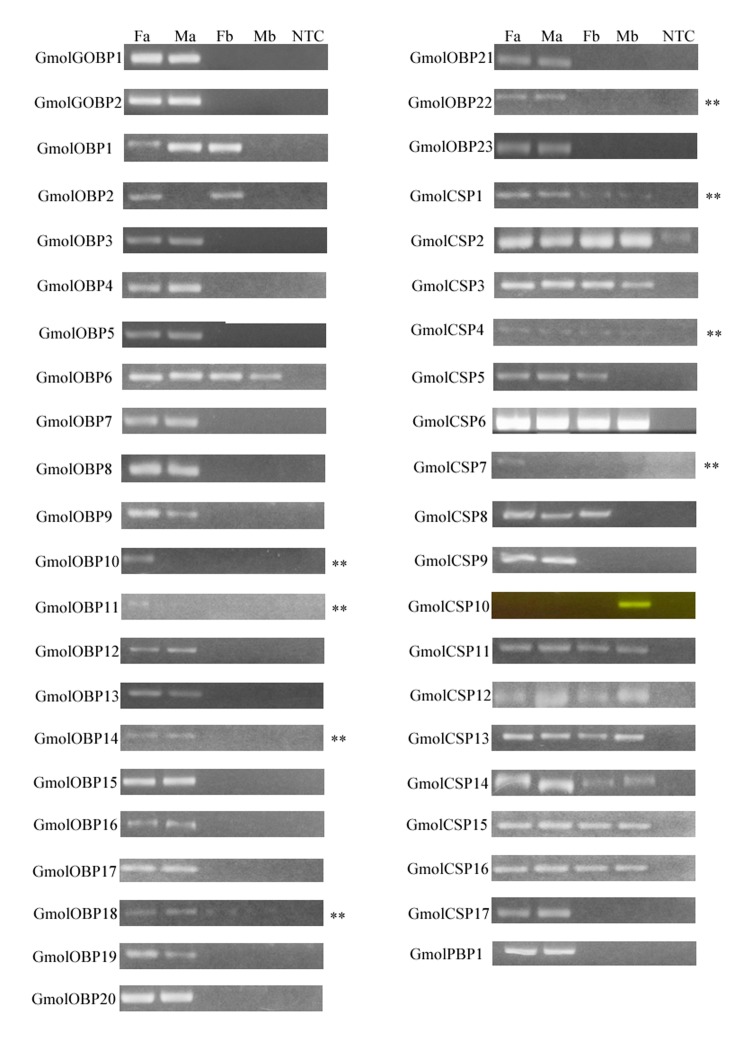
Tissue and sex specific expressions of *G*. *molesta* OBPs and CSPs. Fa: female antennae, Ma: male antennae, Fb: female boby (without antennae), Mb: male boby (without antennae), NTC: No template control.

The sex and tissue-specific expression of GmolPBP2 and GmolPBP3 had been studied previously [49]. In this work, the expression patterns of the candidate genes encoding 26 OBPs and 17 CSPs in male antennae, female antennae, and the remaining bodies were analyzed by semi-quantitative reverse transcription PCR (Fig 10). The results indicated that these OBP-encoding genes were expressed exclusively in antennae except for GmolOBP1, GmolOBP2, GmolOBP6, and GmolOBP18. Interestingly, GmolOBP2 was expressed in both female antennae and the remaining body; whereas GmolOBP10 and GmolOBP11 showed an antenna-specific expression in females. In addition to the expression in both male and female antennae, GmolOBP1 was also expressed in the female body. The remaining OBPs were expressed with similar levels in the antennae of both sexes. Compared with OBPs, almost all candidate CSPs were expressed in the antennae and body of both sexes, appearing no significant differences between males and females.

## Discussion

We used antennal transcriptomic sequencing to identify patutive olfactory genes and identified genes encoding 28 OBPs, 17 CSPs, 48 ORs, four GRs, 24 IRs, two SNMPs and one ODE in female antennae of *G*. *molesta*. The olfactory system genes identified in this work were comparable to recent reports from *H*. *armigera* (47 ORs, 12 IRs, 26 OBPs, and 12 CSPs), *C*. *suppressalis* (47 ORs, 20 IRs, 26 OBPs, 21 CSPs, and 2 SNMPs), *M*. *sexta* (47 ORs, six IRs, 18 OBPs, and 21 CSPs), and *C*. *pomonella*. (43 ORs, 15 IRs, and 1 GR) [3,14,16,17]. Our transcriptome data set appears quite comprehensive since all of the previously annotated *G*. *molesta* olfactory genes available in NCBI were identified in the present antennal transcriptome.

The alignment of the predicted GmolOBPs showed low sequence identity among OBP family members (Fig 4). The predicted proteins have molecular masses ranged between14 to18 kDa. All putative proteins have a signal peptide sequence in the hydrophobic N-terminus. According to the standard established by Hekmat-Scafe [43], insect OBPs can be classified into classical OBPs and atypical OBPs. The six-cysteine signature is the most typical feature of classical insect OBPs [50,51], including GOBP/PBP, CRLBP, ABPI and ABPII. Atypical OBPs families include Minus-C (missing C2 and C5) and Plus-C (carry additional conserved cysteine located between C1 and C2 and after C6). In our work, the classical GmolOBPs were named PBP, GOBP and other OBPs, since the spacing pattern of conserved cysteine in these typical OBPs is C1-X25–68-C2-X3-C3-X31–46-C4-X8–29-C5-X8-C6 (where X is any amino acid). There is only one Minus-C OBPs (named GmolOBP14) that is missing the second and the fifth cysteine. All of the sequences were clustered into GOBP1, GOBP2, PBPs and other OBPs clades in the phylogenetic tree. PBPs are a subfamily of OBPs and constituted of three members in Lepidopteran, PBP1, PBP2, and PBP3. GmolPBP2 (accession number: KF365878) and GmolPBP3 (accession number: KF365879) had already been logged in NCBI database. Another PBP gene was identified by GO annotation and alignment analysis in our antennal transcriptome. We named the unigene Comp42972_c0_seq8 as GmolPBP1, since the coding region was structurally similar to GmolPBP2 and GmolPBP3, although slightly longer than the latter two PBPs. Recent studies have shown that the PBPs subfamily of proteins mainly bind to sex pheromones. Fluorescence binding assays showed that GmolPBP2 had strong binding affinities with (Z)-8-dodecenyl acetate (Z8-12:Ac) and (E)-8-dodecenyl acetate (E8-12:Ac), and the binding constants were 1.09 and 1.10 μmol/L, respectively. The affinity of GmolPBP3 to both main sex pheromones was very weak, the binding constants was only 19.32 μmol/L for Z8-12:Ac and 22.70 μmol/L for E8-12:Ac [49]. Silkworm BmorPBP1 is capable of enhancing sensitivity and selectively mediating the response to bombykol [52]. Bollworm HarmPBP1 binds strongly with two principal pheromone components (Z)-11-tetradecenal and (Z)-9-hexadecenal [53], but the HarmPBP2 and HarmPBP3 showed only weak affinities with the tested ligand. It seems that HarmPBP1 plays a key role in sex pheromone recognition. In *A*. *pernyi* and *A*. *polyphemus*, the binding constants value of PBP1 for principal pheromone component E6,Z11-hexadecadienyl acetate was 1.83 and 0.63 μmol/L, respectively [54]. These results illustrated that the insect PBP1 was the most important pheromone binding proteins. Thus, the affinity of GmolPBP1 with sex pheromone was worth studying.

GOBPs are a subfamily of OBPs, consisting of two members, GOBP1 and GOBP2 in most Lepidopterans. But in tortricid moths and the codling moth (*Cydia pomonella*), which are closely related to *G*.*molesta*, three different transcripts were found to encode putative GOBPs. The GOBPs subfamily can be divided into GOBP1, GOBP2 and GOBP3 [34]. The sequence, which was identified in our antennal transcriptome sequencing and homology-based cloning in female antenna, was named GmolOBP3 (accession number:KF395363), sharing 99% identity with CpomGOBP3 and clustered into GOBPs clade in neighbor-joining tree (Fig 5). Phylogenetic analysis of GmolOBP3 protein showed orthology with GOBPs subfamily genes and probably had similar functions to other GOBP members. GOBPs show spatial specificity in expression, and are localized mainly in adult female and male antennae in Lepidopteran [52]. CpomGOBP3 was detected in antennae, late stage pupal heads, mouthparts and female abdomen tips. It has been speculated that CpomGOBP3 might have a role in oviposition and pheromone production or release, in addition to chemosensation. The GmolOBP3 was only highly expressed in male and female antennae. It has also been hypothesized that GmolOBP3 had a potential role in binding pheromones and plant general odor molecules, and these potential specialized functions in *G*.*molesta* will need to be addressed in future studies.

The tissue expression patterns of the 26 putative OBPs in *G*. *molesta* may help to characterize the function of these OBPs in future research. In this study, semi-quantitative reverse transcription PCR was used to evaluate tissue and sex specific expression levels and abundance of the identified OBPs. Except for GmolPBP2 and GmolPBP3, 22 of the 26 identified OBPs displayed highly antenna-biased expression. The other four genes, HarmOBP1, HarmOBP2, HarmOBP6 and HarmOBP18, were not only highly expressed in antennae, but also expressed equally highly in the remaining bodies. In *A*. *mellifera*, only 9 of 21 OBPs are antenna-specific, and the remaining genes are either expressed ubiquitously or are strictly regulated in specialized tissues or during development. Many reports suggest that OBPs are expressed in taste tissues [55,56], and these genes may play an important role in tasting function and gustatory reorganization.

CSPs were highly and almost ubiquitously distributed in olfactory tissues as well as in non-olfactory tissues, suggesting that CSPs in insects may also participate in other functions in addition to chemosensation [57], such as limb regeneration in *Periplaneta Americana* [58], female survival and reproduction in *Spodoptera exigua* [59], embryo development in *Apis mellifera* [60], migratory behavioral in *Locusta migratoria* [61]. Almost all deduced protein sequences have the characteristic features of CSPs: the presence of a signal peptide and the highly conserved four cysteine profiles (Table 4, Fig 6). Twenty-two putative CSPs have been annotated in *B*. *mori* [16], 21 in *M*. *sexta* [14], 12 in *H*. *armigera* [3], and 21 in *C*. *suppressalis* [17], while we identified 17 candidate CSPs is quite reasonable. Interestingly, tissue- and sex-specific expression demonstrated that GmolCSP7, GmolCSP9 and GmolCSP17 are likely antenna-specific; and these genes perhaps have special roles in detection and transduction of host plant odors molecules.

In *G*. *molesta*, a previously neuroanatomical and computational study found that 48–49 ordinary glomeruli and one large glomerulus situated at the entrance of the antennal nerve in males, and 49–52 ordinary glomeruli and one large glomerulus in the ventro-medial part of the antennal lobe in females [62]. Considering the one receptor-one glomerulus paradigm [63], by which the number of expected ORs in a given species should correlate with the number of glomeruli in the antennal lobe (meaning that one olfactory receptor type is expressed in OSN type), our OR dataset of 48 sequences indicates that there is at least 48 OSN types. These numbers are comparable to the reported numbers in *M*. *sexta* [14], *C*. *pomonella* [17] and *H*. *armigera* [3]. Phylogenetic analysis of the *G*. *molesta* ORs, four of them grouped into a conserved clade containing lepidopteran pheromone receptors (PRs) (Fig 8), and we thus speculate that some or all of them are involved in pheromone recognition. The female *G*.*molesta* moths emit four pheromone compounds, (Z)-8-dodecenyl acetate, (E)-8-dodecenyl acetate, (Z)-8-dodecenyl alcohol and dodecanol [64]. However, the OSNs which are involved in detection of these compounds have not been found in this study. Functional analyses of candidate ORs are usually performed by using heterologous expression in *Xenopus* oocytes and electrophysiology. A distinct receptor of silkworm moth *Bombyx mori*, BmOR3, is expressed in the second ORN, and binds pheromone compound bombykal can inhibit male behavioural response [65]. Candidate pheromone receptors of tobacco budworm *Heliothis virescens* (HvORs), HvOR6 was found to be highly tuned to pheromone (Z)-9-tetradecenal, while HvOR13, HvOR14 and HvOR16 showed specificity for (Z)-11-hexadecenal, (Z)-11-hexadecenyl acetate and (Z)-11-hexadecen-1-ol, respectively [66]. A honey bee *Apis mellifera* ORs, AmOR11 responded specifically to the main “queen substance” component 9-oxo-2-decenoic that maintains the queen’s dominance in the colony and also acts as a long-distance sex pheromone [67]. The *Drosophila* OR, Or43a, has been found that only four odors molecules (cyclohexanol, cyclohexanone, benzaldehyd, and benzyl alcohol) can activate the receptor at a low micromolar concentration, as demonstrated using two-electrode voltage-clamp recording [68]. Electroantennogram (EAG) had illustrated that *G*. *molesta* more sensitive to sex pheromone than females, Z8-12:Ac elicited the strongest antennal response in male [49]. Up to now, the functional of pheromone receptors of *G*. *molesta* is lacking.

The gustatory receptors we identified here, including GmolGR2, GmolGR3 and GmolGR4, were found in a clade with putative sugar receptors (Fig 8). This clade includes the newly characterized *B*. *mori* fructose receptor (BmorGR9) and inositol receptor (BmorGR8) [39,47]. In addition, other identified gustatory receptors (SlitGR4, SlitGR5 and CpomOR25) in moth antennae were also clustered in this family [15,16]. Sugars and other carbohydrates have been shown to influence host preference and oviposition in codling female moths [69]. An artificial mixture of six metabolites of apple, including the three sugar alcohols sorbitol, quebrachitol, and *myo*-inositol and three sugars glucose, fructose, and sucrose, did stimulate the laying of eggs of codling female moths. Fructose, sorbitol and *myo*-inositol are important components of the stimulatory blend. The mated female of *G*. *molesta* prefer egg-laying to surfaces of ripe apple or peach fruit or secrete high carbohydrate on immature fruit. The function of these gustatory receptors seemed related to the recognition of these carbohydrates. In addition, one putative GR receptor (GmolGR1) was identified as a putative CO_2_ receptor, and the protein shares high sequence identity (79%) with the *S*. *Iittoralis* CO_2_ receptor, SlitGR3 [15]. Until this study, moth sensory neurons specific for CO_2_ have been described only on labial palps [36].

Ionotropic receptors represent a novel member of the chemosensory receptor family, which were first discovered in *D*. *melanogaster* by bioinformatics screen genomic data for insect-specific genes with enriched expression in OSNs [11]. These were then found in several other species using genome analyses and antennal transcriptome sequencing. The ionotropic receptor is a variant of the iGluR subfamily. Animal iGluRs have been best characterized for their essential roles in synaptic transmission as receptors for the excitatory neurotransmitter glutamate [70]. In *D*. *melanogaster*, 66 IRs were identified, 15 of which proved to be antenna-specific [11]. Twelve IRs were identified in the antennae of *S*. *littoralis* [15], 15 IRs in the antennae of *C*. *pomonella* [16], 20 IRs in the antennae of *C*. *suppressalis* [17], and 12 IRs in the antennae of *H*. *armigera* [3]. In our study, we found 24 putative IRs in *G*. *molesta* antennae, including two co-receptors, IR8a and IR25a. Compared to ORs, the IR family is relatively conserved in sequence. Among the 24 GmolIRs we discovered, GmolIR8a, GmolIR25a, GmolIR21a, GmolIR41a, GmolIR68a, GmolIR87a, GmolIR93a, GmolIR75d, GmolIR75p1, GmolIR75q.1, GmolIR75q.2, GmolIR76b, GmolIR7d, GmolIR1, and GmolIR3 were clustered in separate clades in neighbor-joining tree with Amel/Bmor/Cpom/Dmel/Msex/Slit/Tcas IRs, respectively. Considering the relatively high sequence conservation and similarities in expression, the functions of GmolIRs are probably conserved as IRs in other Lepidopterans.

## Conclusion

The main purpose of this study was to identify the genes involved in the reception, processing, and degradation of volatiles by analyzing the antennal transcriptome sequence from *G*. *molesta*. The number of OBPs, CSPs, ORs, IRs, GRs, and SNMPs genes that were identified in this species are close to the complete repertoire of olfactory genes from the antennae identified from other Lepidopteran species. The results demonstrated that Illumina Miseq sequencing was successful in the recovery of low-expressing putative olfactory genes, especially in a non-model pest species without an available genome sequence. Our findings made it possible for future research on the molecular level of olfactory system of *G*. *molesta*, and in particular, the discovery of receptor genes will also contribute to the identification of novel volatile host compounds, which would gain new options for controlling insects by mass trapping or disruption.

## Supporting Information

S1 File Amino acid sequences of OBPs, CSPs, ORs, IRs, SNMPs and ODE were used in phylogenetic analyses. (TXT)

S2 File The nucleotide sequences of candidate olfactory genes identified in this study, FASTA formatted file. (TXT)

S1 Table Primers for semi-quantitative reverse transcription PCR expression analyses of *G*. *molesta* OBPs and CSPs. (XLSX)

S2 Table The gene length, reads number, expression level, GO annotation, and BLAST best hit of each unigene. (XLSB)

S3 Table Cover percent of transcripts annotated to putative olfactory genes among the three assemblers. (XLSX)
